# Diversity and Relatedness Enhance Survival in Colour Polymorphic Grasshoppers

**DOI:** 10.1371/journal.pone.0010880

**Published:** 2010-05-28

**Authors:** Sofia Caesar, Magnus Karlsson, Anders Forsman

**Affiliations:** School of Natural Sciences, Linnaeus University, Kalmar, Sweden; University of Zurich, Switzerland

## Abstract

Evolutionary theory predicts that different resource utilization and behaviour by alternative phenotypes may reduce competition and enhance productivity and individual performance in polymorphic, as compared with monomorphic, groups of individuals. However, firm evidence that members of more heterogeneous groups benefit from enhanced survival has been scarce or lacking. Furthermore, benefits associated with phenotypic diversity may be counterbalanced by costs mediated by reduced relatedness, since closely related individuals typically are more similar. Pygmy grasshoppers (*Tetrix subulata*) are characterized by extensive polymorphism in colour pattern, morphology, behaviour and physiology. We studied experimental groups founded by different numbers of mothers and found that survival was higher in low than in high density, that survival peaked at intermediate colour morph diversity in high density, and that survival was independent of diversity in low density where competition was less intense. We further demonstrate that survival was enhanced by relatedness, as expected if antagonistic and competitive interactions are discriminately directed towards non-siblings. We therefore also performed behavioural observations and staged encounters which confirmed that individuals recognized and responded differently to siblings than to non-siblings. We conclude that negative effects associated with competition are less manifest in diverse groups, that there is conflicting selection for and against genetic diversity occurring simultaneously, and that diversity and relatedness may facilitate the productivity and ecological success of groups of interacting individuals.

## Introduction

Studies of animal colour pattern polymorphisms have played an important role in generating and testing hypotheses central to the ecology and evolution of biological diversity both historically [1] and currently [2]. Evolutionary theory predicts that different resource utilization and behaviour by alternative phenotypes may reduce competition and enhance productivity and individual performance in polymorphic, as compared with monomorphic, groups of individuals [3], [4], [5], [6], [7],[8],[9]. Despite a growing interest in the ecological and macro-evolutionary consequences of phenotypic variation and polymorphism [3], [5], [7], [8], [9], [10], little experimental evidence is available to confirm that productivity and individual performance is higher in groups of mixed phenotypes. If different phenotypes represent different genotypes, then any benefits mediated by resource subdivision and reduced competition in groups of more diverse phenotypes may come at a cost of reduced relatedness, since closely related individuals typically are more similar. Relatedness may affect overall levels of cooperative and harmful behaviours, and individuals may act differently in interactions with kin *versus* non-kin [11], [12]. Relatedness may even influence survival if antagonistic behaviours are aimed at relatively unrelated individuals, as in the extreme cases when individuals preferentially kill and eat less related members of the same species [13], [14]. If operating simultaneously, the positive effects of phenotypic diversity and relatedness may result in conflicting selection on genetic diversity. This may translate into differential ecological success among groups with different genetic diversity and determine the fitness benefits of polyandry (i.e., female multi-male mating behaviour) [15], [16], [17], [18], [19].

**Figure 1:**
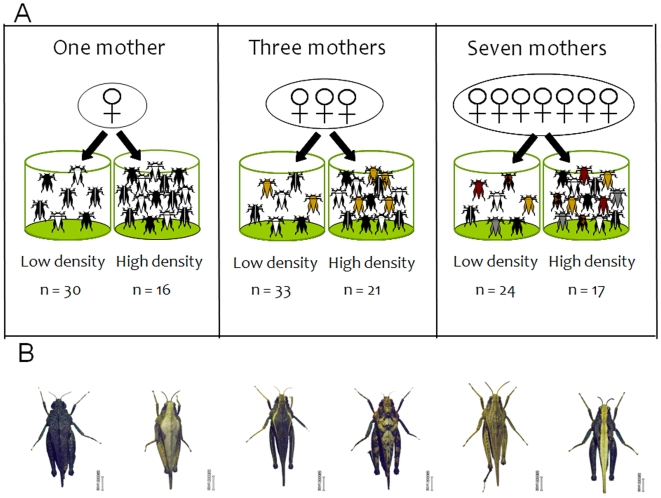
Experimental design used to test for effects of relatedness, density and diversity on survival. (**A**) Newly hatched pygmy grasshoppers (*Tetrix subulata*) from one, three or seven different mothers were placed in experimental cages in high (14 individuals/cage) or low (7 individuals/cage) density. Cages were surveyed on two occasions to determine colour morph diversity and survival of individuals. *n* = numbers of replicates for each treatment. Despite differential survival, the density of individuals was higher in the high (10.1±2.89 individuals/cage) than in the low (5.3±1.27 individuals/cage) density treatment at the time of the first survey in mid July (*F*_1,139_ = 181.48, *P*<0.001). (**B**) Photo showing examples of different pygmy grasshopper colour morphs. Photo: A. Forsman.

Pygmy grasshoppers (Orthoptera: Tetrigidae) exhibit a multitude of discrete colour morphs within populations [20], [21] **(** **Figure 1** **)**, and provide a good model system to explore the roles of diversity and relatedness. The variable colour patterns seem to be controlled by a number of closely linked genetic factors located on one pair of chromosomes [21], a pattern predicted correlated selection [22], [23] as well as by social heterosis favouring alternative haplotypes [8]. Individuals with different combinations of coloration, physiology, and behaviour differently utilize environmental resources and shuttle between microhabitats at very small spatial scales to meet their specific demands regarding body temperature regulation, moisture, perches, food and protection from predators, such that they occupy on the average different niches [24], [25]. Female pygmy grasshoppers mate with multiple males and polyandrous mothers may produce half-sibling offspring that are more colour morph diverse [15], [21], such that sibships vary in genetic relatedness as well as in ecologically important phenotypic diversity. There is circumstantial evidence indicating that survival of offspring may be enhanced by high phenotypic diversity [15] and impaired by reduced relatedness within families [26]. While these results are what one would expect if resource utilization is more efficient and competition less manifest in polymorphic groups [3], [6], [7], [9], [27], there exists as yet no firm evidence that individuals in phenotypically more heterogeneous groups benefit from enhanced survival mediated by reduced competition. The colour polymorphism in pygmy grasshoppers provides a good model system for finding such evidence.

We combined offspring from different numbers of *Tetrix subulata* pygmy grasshopper mothers in experimental groups and tested for effects of phenotypic diversity (colour polymorphism) and relatedness on survival of individuals maintained in high or low density **(** **Figure 1** **)**. We used different densities because we predict effects of diversity to be more pronounced under conditions that impose more intense competition. According to the social heterosis hypothesis, overall productivity may be higher in genetically more diverse groups even in the absence of kin recognition [8], [28]. However, for relatedness to enhance survival via modified levels of antagonistic or cooperative interactions, individuals must be able to discriminate between and behave differently towards kin *versus* non-kin [11], [12], [14], [29]. We therefore compared the behaviour of captive reared individuals that were exposed to scent marks from either a sibling or a non-sibling to examine if pygmy grasshoppers can discriminate siblings from non-siblings based on olfactory cues. Finally, we performed staged encounters and compared inter-individual distances between pairs of siblings and non-siblings to examine if individuals responded differently in interactions with sibling *versus* non-siblings.

Overall, our results support the prediction from theory that individuals in phenotypically more heterogeneous groups benefit from enhanced survival mediated by reduced competition. Survival was also enhanced by relatedness, as expected if co-operative and antagonistic interactions are distributed in favour of closer relatives, and staged encounters confirmed that individuals responded differently in interactions with siblings *versus* non-siblings. Our study demonstrates that negative effects associated with competition are less manifest in diverse groups, that there is conflicting selection for and against genetic similarity occurring simultaneously, and that diversity and relatedness may facilitate the productivity and ecological success of groups of interacting individuals.

## Results and Discussion

**Figure 2:**
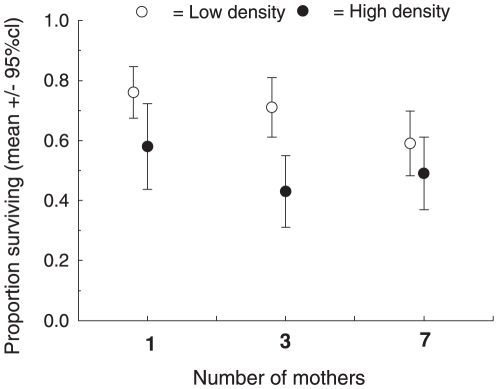
Effects of relatedness on survival of pygmy grasshoppers in high and low density. Survival of captive *Tetrix subulata* from hatching until early autumn as a function of initial density (7 or 14 individuals per cage) and relatedness (number of contributing mothers) of individuals.

To explore if within group diversity enhances the average performance of individuals we combined offspring from different numbers of *Tetrix subulata* pygmy grasshopper mothers in experimental groups and tested for effects of phenotypic diversity (colour polymorphism) and relatedness on survival of individuals maintained in high and low density **(** **Figure 1** **)**. If negative effects of competition and antagonistic interactions are manifested more strongly under crowded conditions then the grasshoppers reared in the high density treatment should experience lower survival as compared with grasshoppers reared in low density. As expected we found that survival from May until mid-September was lower among individuals maintained in high than in low density (logit-model analysis: χ^2^ = 44.89, d.f.  = 1, *P*<0.0001)(**Table 1**, **Figure 2**).

**Table 1:** Effects of density (high versus low) and relatedness (1, 3 or 7 different mothers) on survival of pygmy grasshoppers from hatching in May to end of September.

Source of variation	d.f.	χ^2^	*P*
Density (D)	1	44.89	0.0001
Relatedness (R)	2	15.87	0.0004
D * R	2	6.35	0.0418
*Data for low density*			
Relatedness	2	11.24	0.0036
*Data for high density*			
Relatedness	2	11.36	0.0034

**Figure 3:**
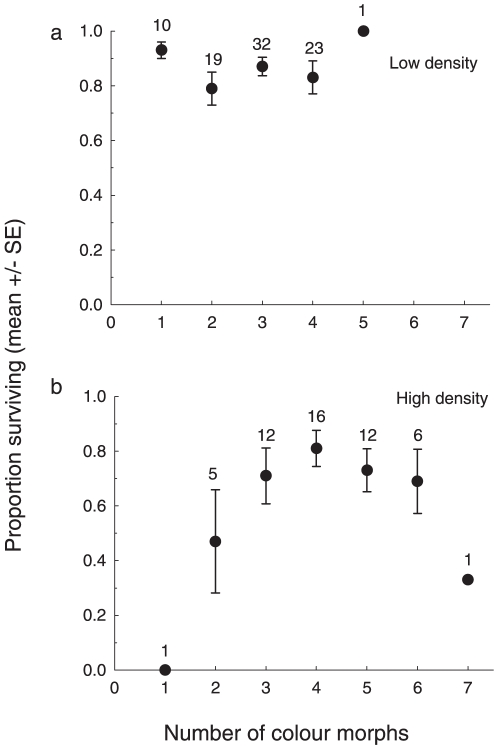
Within-group phenotypic diversity influences survival of pygmy grasshoppers in high but not in low density. Effect of phenotypic diversity (measured as number of colour morphs available in mid July) and density (7 or 14 individuals introduced per cage) on survival from mid July to end of September of captive *Tetrix subulata* maintained in cages at either (**A**) low density or (**B**) high density. Numbers indicate number of replicates in each colour morph category.

If colour morphs with different trait value combinations can avoid competition by utilizing on the average different resources then survival should increase with increased within-group diversity [3]. A positive effect of diversity on survival mediated via niche partitioning should also be expressed more strongly under high density conditions that impose more intense competition for resources [7], [30]. Only coloration was assessed in this study, but it is known from previous work that alternative colour variants of *Tetrix* are eco-morphs that differ in morphology, physiology, life-history, behaviours and microhabitat utilization [24], [25], [31]. Our present results therefore were in agreement with both of the above predictions. The effect of diversity differently affected survival in high and low density (as evidenced by significant effects of the interaction between density and diversity, **Table 2**). In low density, survival from the first census in mid July to the termination of the experiment in late September was not influenced by colour morph diversity (linear effect: χ = 1.34, d.f.  = 1, *P* = 0.25; non-linear effect: χ^2^ = 1.73, d.f.  = 1, *P* = 0.19)(**Table 2**, **Figure 3**). In high density, however, survival increased to an optimum at an intermediate level of diversity of four colour morphs and then declined again (linear effect of number of colour morphs: χ^2^ = 24.13, d.f.  = 1, *P*<0.0001; non-linear effect: χ^2^ = 21.09, d.f.  = 1, *P*<0.0001)(**Table 2**, **Figure 3**). The effect of diversity on survival in high density did not differ among relatedness treatments (effect of the Relatedness * Morphs (linear) interaction: χ^2^ = 2.82, d.f.  = 2, *P*<0.24; Relatedness * Morphs (nonlinear): χ^2^ = 3.42, d.f.  = 2, *P*<0.18). The effect of diversity on survival in high density remained significant also when the two outliers with 1 or 7 colour morphs were omitted from the analysis (effect of the Relatedness: χ^2^ = 5.65, d.f.  = 2, *P*<0.24; Morphs (linear): χ^2^ = 13.90, d.f.  = 1, *P*<0.0002; Morphs (nonlinear): χ^2^ = 10.50, d.f.  = 1, *P*<0.0012).

**Table 2:** Effects of density (high versus low), relatedness (1, 3 or 7 different mothers) and number of colour morphs on survival of pygmy grasshoppers from first census in mid July to end of September.

Source of variation	d.f.	χ^2^	*P*
Density (D)	1	15.09	**<0.0001**
Relatedness (R)	2	2.43	0.2960
Morphs (M, linear effect)	1	0.56	0.4529
Morphs (M2, nonlinear effect)	1	0.14	0.7094
D * R	2	1.85	0.3973
D * M	1	10.47	**0.0012**
D * M2	1	8.21	**0.0042**
R * M	2	3.06	0.2167
R * M2	2	4.44	0.1089
D * R * M	2	0.71	0.7014
D * R * M2	2	0.52	0.7707
*Data for low density*			
Relatedness	2	14.74	**<0.0006**
Morphs (linear effect)	1	1.34	0.25
Morphs (nonlinear effect)	1	1.73	0.19
*Data for high density*			
Relatedness	2	5.49	0.064
Morphs (linear effect)	1	24.13	**<0.0001**
Morphs (nonlinear effect)	1	21.09	**<0.0001**

That survival did not increase indefinitely but instead peaked at intermediate diversity may reflect an inferior survival of rare specialized morphs that were poorly adapted to the conditions in the experimental cages; by chance alone such morphs (genotypes) occurring in low frequency in the source population would be represented primarily in the most diverse replicates founded by several mothers. Diminishing marginal benefits of diversity have been demonstrated in computer simulations showing that the advantage of increasing genetic variability for resource subdivision depends on the number of loci involved; as the number of loci increases, the effect of resource subdivision on the establishment of an initially rare allele introduced into the population decreases [32], [33]. There is a need for additional empirical and theoretical work to determine whether the “hump shaped” (i.e., unimodal) relationship in our study (**Figure 3b**) with a peak in survival at intermediate diversity, is manifest also in other systems as well as to identify the underlying mechanism, since it may have important implications for management of and productivity in biological systems.

Survival was influenced also by relatedness. As expected if co-operative and antagonistic interactions are distributed in favour of closer relatives [11], [12], average survival of newly hatched individuals from the onset of the experiment in May to the termination of the experiment in September decreased with decreasing within-group relatedness (effect of 1, 3 or 7 contributing mothers: χ^2^ = 15.87, d.f.  = 2, *P* = 0.0004)(**Table 1**, **Figure 2**). The effect of relatedness was evident in both low and high density **(** **Table 1** **)**. The positive influence of relatedness remained when the effect of diversity on survival from first census in mid July to termination of the study was controlled for in low density (χ^2^ = 14.74, d.f.  = 2, *P* = 0.0006), but fell short of statistical significance in high density (χ^2^ = 5.49, d.f.  = 2, *P* = 0.064)(**Table 2**).

**Figure 4:**
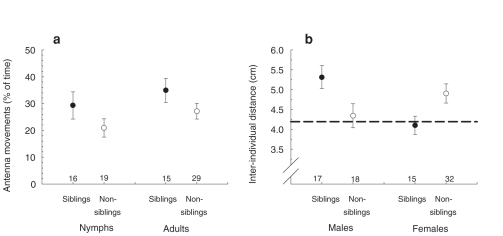
Pygmy grasshoppers respond differently to sibling versus non-sibling individuals. (**A**) Antenna movement activity by pygmy grasshopper nymphs and adults exposed to scent from siblings *versus* non-siblings. Figure shows percentage of time (60 sec) that the focal individual moved its antenna. (**B**) Inter-individual distances in an 8.5 cm diameter Petri-dish for different pairs of interacting pygmy grasshopper individuals that are either siblings or non-siblings, with separate values for males and females. The dotted horizontal reference line represents the average inter-individual distance computed for 1000 pairs of randomized positions along the periphery of an 8.5 cm diameter circular arena. Figures show mean±1 SE. Numbers indicate sample size in each category.

For relatedness to enhance survival via modified interactions individuals must be able to discriminate between and behave differently towards kin *versus* non-kin [11], [12]. One way by which relatedness may influence survival is if individuals preferentially kill less related conspecifics [13], [14]. Behavioural observations suggested that pygmy grasshoppers can distinguish siblings from non-sibling individuals based on olfactory cues: individuals that were exposed to scent marks from a sibling showed a higher rate of antenna movement activity compared with individuals exposed to scent marks from a non-sibling (GLMM, effect of relatedness: *F*_1, 75_ = 4.37, *P* = 0.04; effect of age: *Z* = 6.12, *P*<0.0001)(**Figure 4a**). Since individuals seem to be able to recognize siblings form non-siblings this allows for differential behavioural responses, such as cooperation, active avoidance or aggression and physical interactions. Indeed, our experiment based on staged encounters between pairs of individuals, further revealed that relatedness influenced inter-individual distances: males maintained longer distances to sibling than to non-sibling individuals (GLMM, effect of relatedness: *F*_1, 33_ = 5.34, *P* = 0.027), whereas females kept shorter distances to siblings than to non-siblings (*F*_1, 33_ = 4.27, *P* = 0.045)(**Figure 4b**). Direct physical contact was more common in staged encounters between females (47%, 22 of 47 trials) than males (23%, 8 of 35 trials) (Likelihood ratio χ^2^ = 4.96, d.f.  = 1, *P* = 0.026). In females, physical contact was nearly twice as common in encounters between siblings (66%, 10 of 15 trials) than non-siblings (37%, 12 of 32 trials) (χ^2^ = 4.96, d.f.  = 1, *P* = 0.026). Comparisons with the inter-individual distance expected if individuals were distributed randomly and independently of each other (**Figure 4b**) suggest that the larger inter-individual distances in sibling males and non-sibling females was caused by active avoidance, rather than by attraction or aggression leading to physical contact in non-sibling males and sibling females. That males and females responded differently to relatedness may be explained by their different reproductive roles.

We cannot identify the cause(s) of mortality in our experiment, but that survival was highest in low density **(** **Figure 2** **)** and increased with diversity under high but not under low density **(** **Figure 3** **)** is in accordance with what one would expect if detrimental effects of competition and temporal and micro-spatial subdivision of available resources by alternative morphs were important. Since disruptive selection arising from negative frequency dependent processes may favour rare phenotypes [30], [34], these results imply that niche variation among colour pattern variants may promote the maintenance of polymorphism in pygmy grasshoppers. Other factors that may contribute to the diversity seen in these animals include opposing selection on colour pattern in males and females [35], the polyandrous mating behaviour that results in more diverse offspring and allows for ‘selective free-riders’ that may contribute to the preservation in the next generation also of alternative colour morphs that are not currently favoured by selection [15], divergent selection in combination with gene flow [36], and social heterosis [8], [28], [33].

Since our experimental animals were confined to cages it might be argued that the level of competition and interactions do not reflect natural conditions. However, pygmy grasshoppers produce pods of approximately 30 eggs that hatch synchronously, individuals move only a few meters per day, and nymphs probably remain in close vicinity to where they hatch and interact with siblings and non-sibling individuals, such that our results are applicable to natural conditions [25]. That complementary resource utilization at the micro-spatial scale of our experiment may be important is supported by the finding that resource distributions conspicuous only at the range of centimetres to meters can influence gregarization and have effects at the population level [37]. It has been shown for other species that both genetic and environmental cues may be used to recognize kin from non-kin, and that kin-recognition abilities may disappear when individuals are reared in more variable conditions [29], [38]. It therefore remains an open question whether the effects of relatedness on survival mediated in part by differential behavioural responses to sibling than non-sibling individuals indicated by our results are manifest also among free-ranging individuals under more natural conditions.

In conclusion, we have shown that in groups of pygmy grasshoppers, colour morph diversity and relatedness enhance survival. This supports the proposition that negative effects associated with competition are less manifest in groups of individuals with different capabilities, requirements and tolerances that enable temporal and micro-spatial subdivision of resources, and corroborates one of the mechanisms envisaged to promote the ecological success of polymorphic populations [3], [4], [7], [9]. It is encouraging that the strength in diversity demonstrated in pygmy grasshoppers at the scale of experimental cages mirror associations of variable coloration with endangerment seen at the continent scale across species of lizards and snakes [39] and frogs [40]. Perhaps the most exiting outcome of our study is the demonstration of conflicting selection for and against genetic similarity occurring simultaneously, a finding of importance for models of the evolution of female multi-male mating behaviour [15], [16], [17], [26]. We suggest that future theoretical and empirical research should explore how an enhanced survival of individuals in more heterogeneous groups may be exploited to improve management and restoration of endangered species [41] and increase productivity in biological systems [5], [7], [32].

## Materials and Methods

### Source population and experimental design

Pygmy grasshoppers are small (up to 15-mm body length, 0.07 g), diurnal, ground dwelling insects that exhibit a multitude of discrete colour morphs within populations [20], [21]. Their variable colour patterns seem to be controlled by a number of closely linked genetic factors located on one pair of chromosomes [21]. We collected adult female *Tetrix subulata* in May 2006 from a natural population inhabiting a clear-cut area located in south-central Sweden (N 56°51′ 25″, E 15°35′10″). The site had been intentionally burnt for management and conservation purposes in 2003. We classified females by colour morph and housed them individually for egg-laying (females had mated in the field) in the laboratory. Egg pods were placed on a piece of moist cotton inside a plastic Petri-dish for incubation. More detailed information on the source population, collection procedure, housing conditions and description and photographs of the alternative colour morphs is provided elsewhere [20] and in **Figure 1b**.

To test for independent effects of phenotypic diversity and relatedness on survival we created experimental groups of newly hatched offspring (*N* = 1391 from 91 families) from one, three or seven different mothers (**Figure 1**). We expected the effect of phenotypic diversity on survival to be more pronounced under conditions that impose intense competition for resources, and therefore reared individuals under different density regimes; low (7 individuals/bucket) or high (14 individuals/bucket). A low density one-mother replicate consisted of 7 offspring from the same mother, a three-mother replicate consisted of two offspring from two different mothers plus three offspring from a third mother, and a seven-mother replicate consisted of one offspring each from seven different mothers. A high density one-mother replicate consisted of 14 offspring from the same mother, a three-mother replicate consisted of five offspring from two different mothers plus four offspring from a third mother, and a seven-mother replicate consisted of two offspring each from seven different mothers.

Experimental groups were housed in separate, white 10-l plastic buckets (22 cm diameter) that contained a peat-soil mixture, were supplied with a piece of *Polytricum* moss, maintained out-of-doors and watered at regular intervals to promote the growth of mosses and algae for food [20]. Buckets were covered with fibre–cloth to prevent the animals from escaping while still allowing flow of air, light and water. Individuals used the surface and crevices in the peat-soil substrate, but also climbed the moss and inner walls of the bucket as well as the underside of the fibre-cloth cover to feed and seek out different temperature, light and humidity regimes.

Since it is not possible to reliably determine the colour morph of hatchlings we determined within group diversity (number of colour morphs) by counting and classifying all individuals by colour morph on 17–18 July, when nymphs were about six weeks. Number of colour morphs available within groups increased with number of contributing mothers (*F*_2,136_ = 7.16, *P*<0.01)(**Figure S1**). This variation in diversity among and within kinship treatments enabled us to estimate the direct effect of diversity at first census in mid July on survival from thereon until the termination of the experiment on 20–21 September while controlling statistically for effects of relatedness and density. We do not know how much diversity was lost due to mortality in the first weeks, but despite the mortality during the first part of the experiment the difference in density between treatments was still evident at the time of the first census (**Figure 1**).

### Kin recognition experiment

To examine if pygmy grasshoppers can discriminate between sibling and non-sibling individuals based on olfactory cues we compared the behaviour of captive reared nymphs (*N* = 37) and adult (*N* = 49) individuals that were exposed to scent marks from either a sibling or a non-sibling. Before the trials, test individuals had been reared in singly in separate cages from the day of hatching until the onset of the scent discrimination experiment on 8 October 2007, and were born to females collected 25 April and 9 May, 2007, from the same source population as described earlier. For each replicate, a scent-donor individual was placed on a piece of cotton (moist with distilled water) in a Petri dish for 2 min. The donor was then replaced by either a sibling or a non-sibling that was allowed to explore the dish while we recorded its' behaviour with a video camera. The percentage of time (60 sec, starting 10 sec after release inside the dish) during which the individual actively moved its antenna was computed from video recordings that were labelled with a randomly drawn number and analyzed by a person (C.K.) unaware of experimental treatment. New latex gloves, Petri dishes and pieces of cotton were used for each replicate. Trials were performed in a laboratory at 21±3°C with four fluorescent strip lights (Philips Master TL 28W/830 HE) mounted in the ceiling. Data for 5 adults and 2 nymphs that remained motionless and performed no exploratory behaviour during the 60 sec observation period were omitted from the analyses.

To examine if individuals responded differently in interactions with sibling and non-sibling individuals we compared the inter-individual distance for different pairs of interacting captive reared adult male (*N* = 35 pairs) and female (*N* = 47 pairs) pygmy grasshopper individuals that were either siblings or non-siblings. Before the experimental trials, individuals were housed singly in separate cages. For each replicate, two individuals were placed in a Petri dish (8.5 cm diameter) and their behaviours recorded with a video camera for 3 min. Inter-individual distance was measured every 30 sec, starting ca 40 sec after release inside the dish, from video recordings displayed on a monitor using a metric tape to the nearest 0.5 cm and then converted back to real distances. Recordings were labelled with a randomly drawn number and analyzed by a person (M.F.) unaware of experimental treatment. We used mean values for the 5 observations per pair in the statistical analyses. Trials were performed in a laboratory between 28 April and 12 May 2009. To obtain a reference value expected under the null hypothesis that individuals distributed themselves inside the Petri dish randomly and independently of the other individual, we generated a sample of 1000 inter-individual distances computed from pairs of randomized positions along the periphery of an 8.5 cm diameter circular arena. Since the randomized positions represent grasshopper midpoints we subtracted 2×0.5 cm from each inter-individual distance and then computed the average value (4.19 cm).

### Statistical analyses

We used logit-model analysis suitable for modelling and analyzing binary data in the form of proportions [42] to test for effects of density (7 or 14 nymphs initially released in each bucket), relatedness (one, three or seven mothers) and diversity (number of colour morphs within each group at first census) on survival. Maximum likelihood estimates of the parameter values were computed through an iterative fitting process implemented by the procedure GENMOD [43] and the Wald statistic was used to assess the statistical significance of explanatory variables included in the final model [42]. Relatedness and density were included as categorical factors. Number of colour morphs at first census was included as a continuous variable to test for linear and non-linear effects of diversity. We started with a saturated, most complex model that contained all possible interaction effects and then excluded non-significant explanatory variables through an iterative fitting process. A common variance inflation factor for all observations (computed as the square root of the Pearson's chi-square divided by the degrees of freedom) was used to account for problems associated with overdispersion [43]. Survival of newly released hatchlings to the termination of the experiment at the end of September was influenced by the interaction between density and relatedness (χ^2^ = 6.35, d.f.  = 2, *P* = 0.042), so we performed separate tests for effects of relatedness in high and low density **(** **Table 1** **)**. Similarly, because of significant effects of the interaction between diversity and density on survival from first census to termination of the study (**Table 2** **,** **Figure 3**) we performed separate tests for effects of diversity and relatedness on survival in high and low density. We analyzed data from the kin-recognition experiment with Generalized Linear Mixed Models (GLMMs) implemented in SAS [44]. Arcsine square-root transformed proportion of total time (60 sec) spent moving the antenna was the dependent variable, relatedness a fixed factor and age (nymph vs. adult) a random factor. In the analyses of data on inter-individual distances both relatedness and sex was treated as fixed factors. Since there was an effect on inter-individual distances of the two-way interaction between relatedness and sex (GLMM: *F*_1, 78_ = 9.64, *P* = 0.0027) we performed separate tests for effects of relatedness in males and females.

## Supporting Information

Figure S1 Phenotypic diversity (measured as number of different colour morphs available at first census in mid July) in experimental groups founded by few or many hatchling Tetrix subulata produced by different numbers (1, 3 or 7) of contributing mothers. Figure shows mean+/− s.e.
(0.02 MB EPS)
